# Strain-Tunable Electronic Properties and Band Alignments in GaTe/C_2_N Heterostructure: a First-Principles Calculation

**DOI:** 10.1186/s11671-018-2708-x

**Published:** 2018-09-26

**Authors:** Xiao-Huan Li, Bao-Ji Wang, Xiao-Lin Cai, Wei-Yang Yu, Ying-Ying Zhu, Feng-Yun Li, Rui-Xia Fan, Yan-Song Zhang, San-Huang Ke

**Affiliations:** 1 School of Physics and Electronic Information Engineering, Henan Polytechnic University, 2001 Shiji Road, Jiaozuo, 454000 China; 2 MOE Key Labortoray of Microstructured Materials, School of Physics Science and Engineering, Tongji University, 1239 Siping Road, Shanghai, 200092 China

**Keywords:** GaTe/C_2_N, Heterostructure, Density functional theory, Strains, Multifunctional devices

## Abstract

Recently, GaTe and C_2_N monolayers have been successfully synthesized and show fascinating electronic and optical properties. Such hybrid of GaTe with C_2_N may induce new novel physical properties. In this work, we perform ab initio simulations on the structural, electronic, and optical properties of the GaTe/C_2_N van der Waals (vdW) heterostructure. Our calculations show that the GaTe/C_2_N vdW heterostructure is an indirect-gap semiconductor with type-II band alignment, facilitating an effective separation of photogenerated carriers. Intriguingly, it also presents enhanced visible-UV light absorption compared to its components and can be tailored to be a good photocatalyst for water splitting at certain pH by applying vertical strains. Further, we explore specifically the adsorption and decomposition of water molecules on the surface of C_2_N layer in the heterostructure and the subsequent formation of hydrogen, which reveals the mechanism of photocatalytic hydrogen production on the 2D GaTe/C_2_N heterostructure. Moreover, it is found that in-plane biaxial strains can induce indirect-direct-indirect, semiconductor-metal, and type II to type I or type III transitions. These interesting results make the GaTe/C_2_N vdW heterostructure a promising candidate for applications in next generation of multifunctional optoelectronic devices.

**Electronic supplementary material:**

The online version of this article (10.1186/s11671-018-2708-x) contains supplementary material, which is available to authorized users.

## Background

Ever since the discovery of graphene [1, 2], interest in two-dimensional (2D) layered materials has been growing steadily. Many graphene-like 2D materials such as transition-metal dichalcogenides [3], monolayer honeycomb structures of group V elements and III-V binary compounds [4–8], and post transition metal chalcogenides (PTMCs)[9] have gained a lot of interest due to their exceptional physical properties and promising applications. Among these diverse 2D materials, GaTe monolayer, as a member of PTMCs [9], has successfully been fabricated by molecular beam epitaxy [10]. Theoretical calculations showed that GaTe monolayer is an indirect-bandgap semiconductor and its bandgap can be modulated by applying strains [11]. Besides, monolayer C_2_N, a new 2D layered material with uniform pore and nitrogen atom distributions, was also successfully synthesized via a bottom-up wet-chemical reaction and found to be a direct-gap semiconductor [12]. Many studies demonstrated that its bandgap, band edge positions, and optical properties can be engineered by varying their stacking order, layer number, external electric field or strain and alloying/substituting with other elements [13–16]. It should be noted that the tunable direct bandgap and porous nature of C_2_N is expected to exhibit desirable properties for electronics, optoelectronics, and energy conversion as well as photocatalytic water splitting, etc [15]. However, a significant challenge still remains for the use of C_2_N in photocatalysis and photovoltaic cells: The photogenerated electron-hole pairs stay in the same regions spatially, which can lead to a high rate of recombination of photogenerated carriers, thus reducing the solar energy conversion

In parallel with the efforts on single 2D materials, the van der Waals (vdW) heterostructures fabricated by stacking different 2D semiconductor materials have opened up new avenues for creating new materials and designing new devices [17–23]. This sort of heterostructure can be generally classified as three types: the type I (straddling gap), type II (staggered gap), and type III (broken gap) according to the relative positions of the valence band maximum (VBM) and conduction band minimum (CBM) of the respective semiconductors [18, 24, 25]. For the type I heterostructures, the energies of the VBM and CBM of one material straddle those of the other material and all the photogenerated electrons and holes are accumulated in the same layer, which induces the ultrafast recombination of the excited carriers and thus can be utilized in optoelectronic devices, such as light-emitting diode. In the case of type II heterostructures, both the CBM and VBM of one material are lower or higher in energy than the ones of the other material. As a result, photogenerated electrons and holes are confined separately in the two materials, respectively, thereby inhibiting the rate of recombination. Therefore, they can be used as building blocks for photovoltaic devices [18, 24]. As for the type III heterostructures, the VBM level of one material is higher than the CBM level of the other, which is desirable for tunneling field effect transistors [25, 26]. Very recently, many GaTe-based heterostructures have been extensively studied both theoretically and experimentally. The GaTe/InSe heterostructure has been fabricated experimentally and presents the type II band alignment [27, 28]. Quasi-2D GaTe/GaSe heterostructure was created by transferring exfoliated few-layer GaSe onto bulk GaTe sheets and found to form type I band alignment at the interface [29]. The GaTe/SnI heterostructure was verified to be a large-gap quantum spin Hall insulator and exhibits a noticeable Rashba splitting that can be modulated by changing the interlayer distance of heterosheets [30]. In addition, construction of semiconductor/C_2_N heterostructures, such as g-C_3_N_4_/C_2_N [31], MoS_2_/C_2_N [32], and CdS/C_2_N [33], demonstrated an enormous potential for promoting the photocatalytic performance of C_2_N due to the efficient separation of the electron-hole pairs, thereby restraining the recombination of photogenerated carriers.

In this work, we construct the GaTe/C_2_N vdW heterostructure and perform first-principles density functional theory (DFT) calculations to investigate its structural parameters and electronic, optical properties. The results show that the heterostructure possesses intrinsic type II band alignment and better visible-UV light absorption than the constituent layers. Moreover, we predict the strain dependences of the bandgap, band alignments, and band edge positions of the GaTe/C_2_N heterostructure, which are essential in the design of new multi-functional nano-devices.

## Methods

In our research, we perform first-principles calculations by using the Vienna ab initio simulation package (VASP) [34]. A plane-wave basis set with a kinetic energy cutoff of 500 eV and Perdew-Burke-Ernzerhofer (PBE) projected augmented wave pseudopotential [35] are adopted to expand the wave functions and to describe the electron-ion potential, respectively. The computationally more expensive hybrid Heyd-Scuseria-Ernzerhof (HSE06) functional method [36] is adopted to correct the underestimated bandgaps obtained by DFT/PBE calculations. The weak vdW interaction between the two monolayers is described by the DFT-D2 correction of Grimme [37]. A vacuum space in the *z*-direction more than 25 Å is used to avoid interactions between adjacent heterobilayers. A 21×21×1 (11×11×1) *k*-mesh for the PBE (HSE06) calculations is utilized to sample the Brillouin zone. The atomic positions are fully relaxed until energy and forces are converged to 10^−5^ eV and 0.01 eV/Å, respectively.

## Results and Discussion

**Fig. 1:**
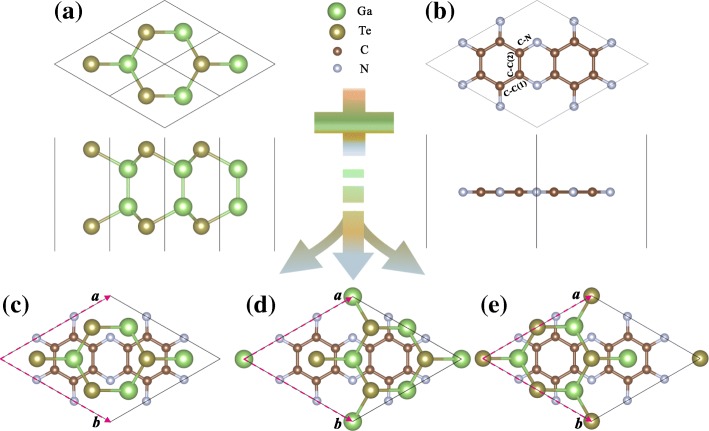
Top and side views of (**a**) GaTe and (**b**) C_2_N monolayers. Top views of (**c**–**e**) *α*-, *β*-, and *γ*-stacking GaTe/C_2_N heterostructures, in which the corresponding base vectors of the heterostructures are labeled

Let us start from the investigations of the pristine GaTe and C_2_N monolayers. The optimized configurations of the two monolayers are shown in Fig. 1a, b, respectively. Their structural parameters are listed in Table 1. For GaTe monolayer, the optimized lattice constant and Ga-Te bond length are 4.14 and 2.41Å respectively. In the case of the C_2_N monolayer, the optimized lattice constant, C-N, and C-C(1)/C-C(2) distances are 8.26, 1.34, and 1.47/1.43Å, respectively. Furthermore, their band structures are also investigated by the PBE/HSE06 calculations and presented in Additional file 1: Figure S1a and b, respectively. Apparently, the GaTe monolayer is a semiconductor with an indirect bandgap of 1.43/2.13 eV while C_2_N monolayer is a direct bandgap semiconductor with a value of 1.65/2.44 eV. Meanwhile, we find that aside from a rigid shift, the band structures of C_2_N monolayer calculated with PBE and HSE06 differ significantly, especially for the valence bands. However, the CBMs and VBMs calculated using PBE and HSE06 are all at *Γ* points, indicating the band dispersions given by the two functionals are relatively consistent though there is some difference in accuracy. All the results are in good agreement with those of previous reports [11, 38] and suggest the reliability of our calculation method. As is well known, bandgaps of semiconductors are generally underestimated by the PBE functional because of the lacking of the derivative discontinuity in the energy functional. Our subsequent presentation for the electronic and optical properties will be based on the HSE06 results.

**Table 1:** The calculated ground-state properties of the monolayers and their heterostructures: lattice parameters of primitive unit cell *a* and *b*, bond lengths, interlayer distances (*d*_il_), interlayer binding energy *E*_b_ (per unit area), and bandgaps calculated within vdW-DFT/PBE $\left (E_{\mathrm {g}}^{\text {PBE}}\right)$ and vdW-DFT/HSE06 $\left (E_{\mathrm {g}}^{\text {HSE}}\right)$

	*a*=*b*	*d*_Ga−Te_	*d*_Ga−Ga_	*d*_C−C(1)/C(2)_	*d*_C−N_	*d*_il_	*E*_b_	$E_{\mathrm {g}}^{\text {PBE}}$	$E_{\mathrm {g}}^{\text {HSE}}$
	(Å)	(Å)	(Å)	(Å)	(Å)	(Å)	(meV/Å^2^)	(eV)	(eV)
GaTe	4.14	2.71	2.47	1.47/1.43				1.43	2.13
C_2_N	8.32			1.46/1.42	1.34			1.65	2.44
*α*-stacking	8.26	2.69	2.43	1.46/1.42	1.33	3.70	− 15.06	0.70	1.42
*β*-stacking	8.26	2.69	2.43	1.46/1.42	1.33	3.71	− 14.97	0.68	1.42
*γ*-stacking	8.26	2.70	2.42	1.46/1.42	1.33	3.50	− 15.80	0.60	1.38

The GaTe/C_2_N heterobilayer is constructed by combining a 2×2 supercell of GaTe sheet and a 1×1 unit cell of C_2_N layer, with the only 0.48% lattice mismatch. In order to find the stable configuration of the heterostructure, we shift the GaTe monolayer in different directions. As a result, three energetically favorable stacking types with high symmetry named as *α*-, *β*-, and *γ*-stacking are obtained, as illustrated in Fig. 1c–e. In the *α*-stacking, the hexagonal C_4_N_2_ rings are right over the hexagonal GaTe rings. As for the *β*- and *γ*-stacking, they can be obtained by moving GaTe layer in the *α*-stacking about 1.21 and 2.42 Å along the ***a*** + ***b*** direction, respectively. To compare the relative stabilities of the three stacking configurations, we calculate their interface binding energies, $\phantom {\dot {i}\!}E_{\mathrm {b}} = (E_{\mathrm {GaTe/C_{2}N}}-E_{\text {GaTe}}-E_{\mathrm {C_{2}N}})/S$, where $\phantom {\dot {i}\!}E_{\mathrm {GaTe/C_{2}N}}$, *E*_GaTe_, and $E_{\mathrm {C_{2}N}}$ represent total energies of the GaTe/C_2_N heterostructure, free-standing GaTe and C_2_N monolayers, respectively, and *S* is the surface area of the 2D supercell. As shown in Table 1, the binding energies of GaTe/C_2_N heterostructures with *α*-, *β*-, and *γ*-stacking configurations are − 15.06 meV, − 14.97 meV, and − 15.80 meV/Å^2^, respectively. The three binding energies are very close to each other though the *γ*-stacking is energetically more favorable, which is consistent with its smallest interlayer distance. We further confirm the dynamic and thermal stabilities of these heterostructures with different stacking forms by calculating their phonon spectra and performing ab initio molecular dynamics (MD) simulations and show the results in Additional file 1: Figure S2. All phonon modes have positive frequencies except for the transverse acoustic mode near the *Γ* point due to the phonon softening, confirming the dynamic stability [5]. In the MD simulations, the total energies of the systems oscillate in certain energy ranges, and no geometric reconstructions and broken bonds are found to occur in the heterostructures, indicating that these systems are thermally stable at room temperature [39]. We note that during MD simulation the *γ*-stacking configuration possesses the least energy undulation (less than 7 meV/atom), indicating its more prominent thermal stability. The very close binding energies of the three stacking configurations implies that their electronic structures may also be very similar. To confirm this, we calculate the band structures for the three configurations (see Additional file 1: Figure S3). One can see that the three band structures are indeed almost identical. Although the *γ*-stacking configuration is the most stable one, the three configurations may still be populated with some probabilities at room temperature because of their similar formation energies. However, because their electronic structures are also very close to each other, we can choose only one configuration to present our work. Here, we choose the most stable *γ*-stacking configuration in the following analyses and discussions.

**Fig. 2:**
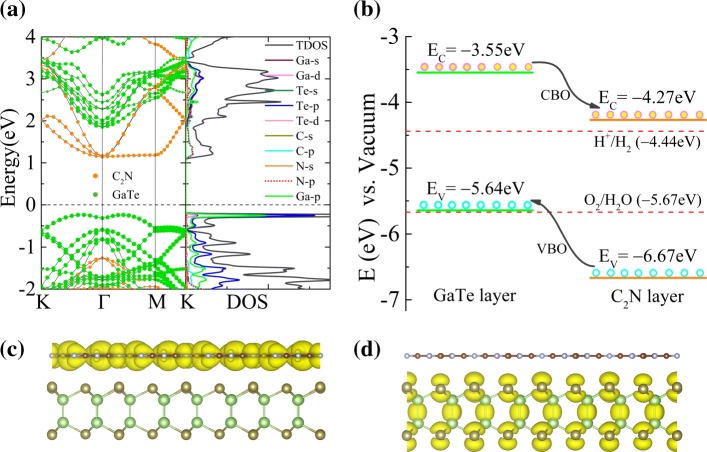
**a** The projected band structure of the GaTe/C_2_N heterostructure with *γ*-stacking configuration and the corresponding total and partial density of states. **b** Schematic representation of type II band alignments for the carrier transfer and separation in the GaTe/C_2_N heterostructure, referring to the vacuum level. The redox potentials (red dashed line) of water splitting at pH =0 are shown for comparison. Band decomposed charge densities of the **c** VBM and **d** CBM of the heterostructure

We now go to the electronic properties of the GaTe/C_2_N vdW heterostructure. As shown in Fig. 2a, the bandgap of GaTe/C_2_N heterostructure is calculated to be about 1.38 eV. In comparison with that of its components, its bandgap is reduced due to the GaTe-C_2_N interaction and the resulting band alignment. Also, the electronic structure of C_2_N monolayer is well-preserved. Nevertheless, the projected band structure of GaTe in the heterostructure have considerable changes comparing to the monolayer, which can be attributed to the fact that the interlayer vdW and electrostatic interactions can result in the overlap of electronic states in the bands of the heterostructure. Similar behavior is also found in MoS_2_/PbI_2_ vdW heterostructure [40]. Furthermore, we find that its VBM and CBM are mainly localized on GaTe and C_2_N sublayers, respectively. From the calculated total and partial density of states (PDOS) in Fig. 2a (right panel), it can be seen that the CBM mainly originates from the *p* states of N and C atoms, whereas the VBM is mainly dominated by the *p* states of Te and Ga atoms. The band decomposed charge densities of the CBM and VBM in Fig. 2c, d reveal that the lowest-energy electrons and holes are distributed in the C_2_N layer and GaTe layer, respectively, consistent with the detailed PDOS results above. The band alignment of the GaTe/C_2_N heterostructure including both VB offset (VBO) and CB offset (CBO) is illustrated in Fig. 2b, which is according to the analysis of Fig. 2a. Clearly, the VB and CB of the GaTe layer are higher in energy than the corresponding bands of the C_2_N layer, and the VBO and CBO between the GaTe and C_2_N layers are about 1.03 and 0.72 eV, respectively. When the heterostructure is illuminated with light, the electrons with energy obtained from the sunlight leap into the CB from the VB. And then these photogenerated electrons on the CB of the GaTe sheet can be easily shifted to that of the C_2_N layer due to the observed CBO. Conversely, the photogenerated holes on the VB of the C_2_N sheet transfer to that of the GaTe layer because of the VBO. The above results suggest that a type II band alignment is formed at the interface between GaTe and C_2_N layers, which is a prerequisite to separate the electrons and holes efficiently. In addition, the calculated plane-averaged charge density difference of the heterostructure, shown in Additional file 1: Figure S4, indicates that some electrons transfer from the C_2_N layer to the GaTe layer. It means that an intrinsic built-in electric field (*E*_in_) is induced with its direction pointing from C_2_N layer to GaTe layer. Also note that the *E*_in_ acts in opposite (same) direction to the transfers of photogenerated electrons (holes) and thus inhibits the recombination of photogenerated electron-hole pairs. As a result, under the combined effect of intrinsic *E*_in_ and band offset, the photogenerated carries can be effectively separated on different surfaces, which can improve the energy conversion efficiency and finally enhance the performance of optoelectronic devices.

Besides, we notice that the CBM of the heterostructure locates more positive than the reduction potential (− 4.44 eV vs vacuum level) of hydrogen evolution, whereas its VBM almost overlaps with the oxidation potential (− 5.67 eV vs vacuum level) of oxygen evolution. Hence, it has only limited photocatalytic capacity to split water by producing hydrogen at pH =0. Nevertheless, changing the inter-layer spacing and pH value can ignite the potential application of the heterostructure as a visible light photocatalyst (see later discussion in details).

**Fig. 3:**
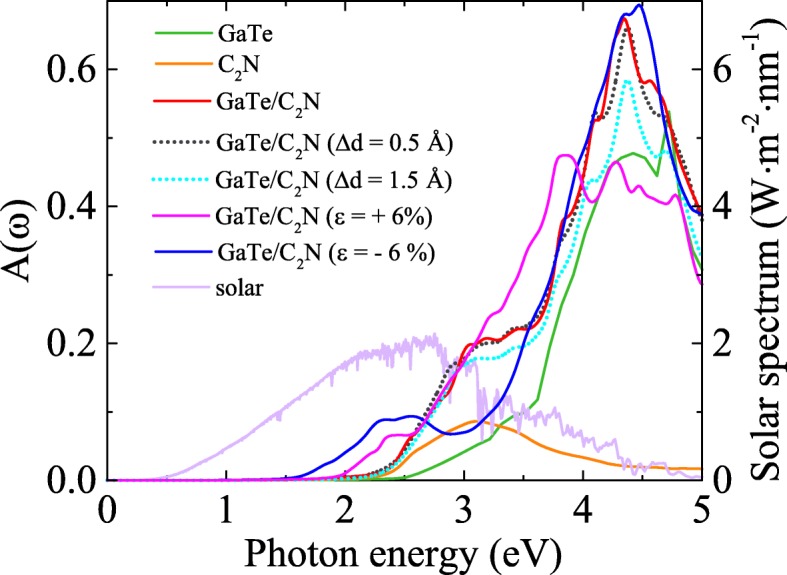
The calculated optical absorption spectra *A*(*ω*) of the GaTe/C_2_N heterostructure and its components using hybrid HSE06 functional. *A*(*ω*) of the heterostructures with vertical strains of 0.5 Å and 1.5 Å and in-plane strains of +6% and -6%. And the solar spectrum are also shown for comparison

Actually, a promising photoelectric nano-device should absorb as much visible-UV light as possible. Thus, we further explore the optical absorptions of the GaTe/C_2_N heterostructure and its components. The computational details have been fully described in our previous works [22, 23]. As displayed in Fig. 3, the GaTe/C_2_N heterostructure exhibits stronger visible-UV light absorption and a wider absorption range compared with its components, especially in the energy range of 2.20 to 4.71 eV. This stems from the new optical transitions induced by the charge transfer and interlayer coupling in the heterostructure [41].

It is widely known that strains, including inter-layer (normal) and in-plane strains, provide an effective way to tune the electronic properties and thus enhance the performance of materials [42]. Here, we first explore the normal strain effect in GaTe/C_2_N vdW heterostructure. The normal strain is evaluated by *Δd*=*d*−*d*_0_, where *d* and *d*_0_ are the actual and equilibrium distances, respectively, between GaTe and C_2_N sublayers. Thus, if *Δd*>0, the system is under a normal tensile strain, and vice versa. The change in the interaction between the GaTe and C_2_N layers should be reflected by the intensity of charge transfer between them. The calculated plane-averaged charge density differences of the GaTe/C_2_N heterostructures with different interlayer distances are shown in Additional file 1: Figure S5. The results show that as the distance between the GaTe and C_2_N sheets decreases, the charge transfer obviously intensifies as a result of the enhanced interlayer interaction. Thus, the electronic behavior of the GaTe/C_2_N heterostructure is expected to be well tuned by normal strain.

**Fig. 4:**
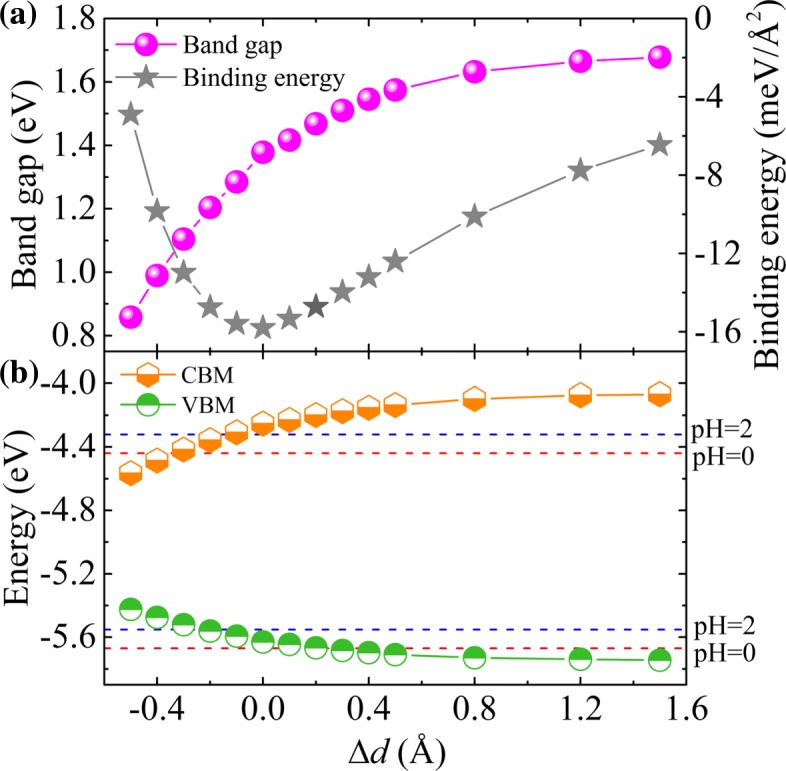
Normal strain effects on **a** the bandgap and biding energy, and **b** the band-edge positions of GaTe/C_2_N vdW heterostructure. The redox potentials of water splitting at pH 0 (red dashed line) and pH 2 (blue dashed line) are shown for comparison

The calculated bandgap and binding energy of the heterostructure as functions of the applied strain are shown in Fig. 4a, and the evolutions of the CBM and VBM of the heterostructure under normal strain are shown in Fig. 4b. It is clearly shown that an increasing normal compressive strain reduces the bandgap due to the enhanced inter-layer interaction. In contrast, an increasing normal tensile strain first increases slowly the bandgap and then reach nearly a convergence at *Δd*≃0.8Å, which can arise from the greater reduction of the inter-layer interaction [32]. We find the equilibrium structure at *Δd* = 0 has the lowest binding energy, which is consistent with the result shown in Table 1. Meanwhile, we notice that the type II band alignments and enhanced visible-UV light absorption are preserved, being nearly irrespective of the inter-layer distance (see Fig. 3 and Additional file 1: Figure S6). More interestingly, the large tensile normal strains (*Δd*≃0.3 Å) shift the VBM below the O_2_/H_2_O oxidation potential, making the system suitable for water splitting at pH = 0. During the photocatalytic water splitting, the hydrogen and oxygen production processes will occur separately in the C_2_N layer and GaTe layer, respectively. We note that under such situation, the VBM over-potential is so small that it may not be sufficient for O_2_ production [43], but such bias potentials can be tuned by changing the pH value of the medium [44]. In other words, the photocatalytic properties for water splitting can be further modulated by controlling the pH to match the redox potential of water. As illustrated in Fig. 4b, in the acidic environment of pH = 2, the band edges of the heterostructure perfectly straddle the water redox potential, showing the heterostructure is well suitable for H_2_/O_2_ production from water, especially for large vertical strains applied.

**Fig. 5:**
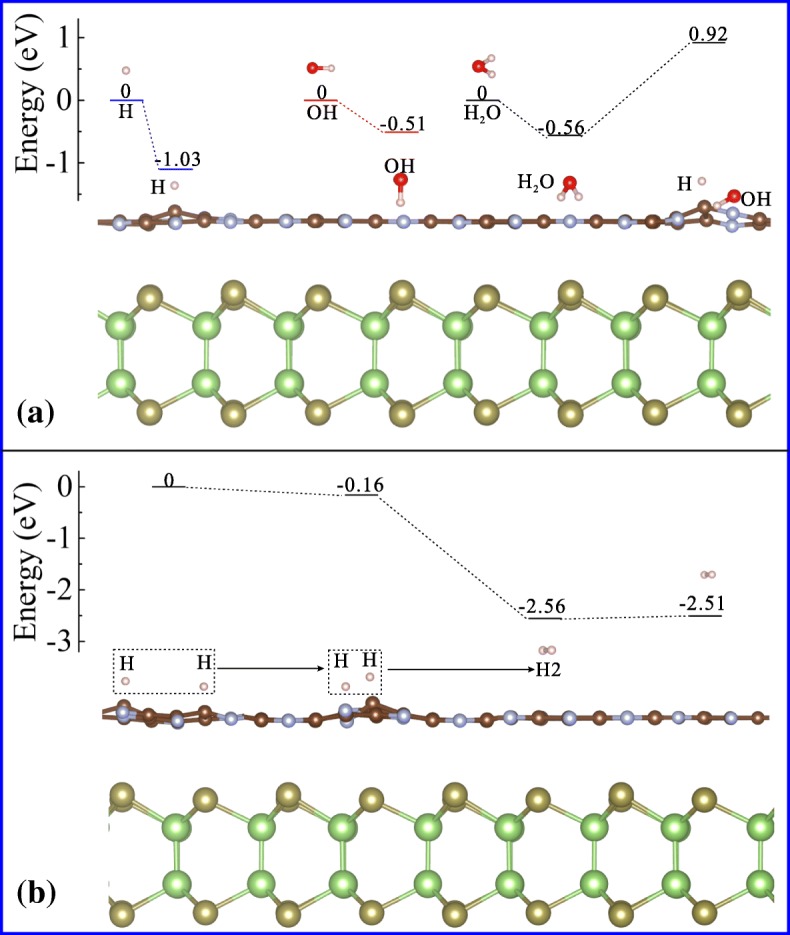
**a** Adsorption configurations of H, OH, H_2_O and decomposition mechanism of H_2_O on C_2_N surface in GaTe/C_2_N vdW heterostructure. **b** Interaction between two hydrogen adatoms, formation and releasing of hydrogen molecular on C_2_N surface in GaTe/C_2_N vdW heterostructure

To further reveal the mechanism of the photocatalytic hydrogen generation on GaTe/C_2_N heterostructure, we simulate the water adsorption and decomposition on the surface of the C_2_N layer, where hydrogen is produced during the photocatalytic water splitting. Since the formation of hydrogen molecules starts from the decomposition of absorbed water, we firstly investigate the absorption energy of H, OH, and H_2_O on the C_2_N surface at the DFT/PBE level. The corresponding adsorption energies are −1.03, −0.51, and −0.56 eV, respectively, as illustrated in Fig. 5a. The negative values indicate that the absorptions are energetically favorable [45]. Subsequently, the calculated reaction energy of water decomposition is about 1.48 eV (from − 0.56 to 0.92 eV). This means that water decomposition is an endothermic reaction on this surface. Furthermore, as the generated hydrogen atoms are adsorbed on C_2_N surface, the remotely separated hydrogen adatom will be energetically favorable to migrate close to form hydrogen molecules [46]. As displayed in Fig. 5b, the reaction energy required for removing one H_2_ from C_2_N is relatively small (0.04 eV), which indicates that the adsorbed H_2_ is easy to be released and is beneficial for photocatalytic hydrogen gas production.

**Fig. 6:**
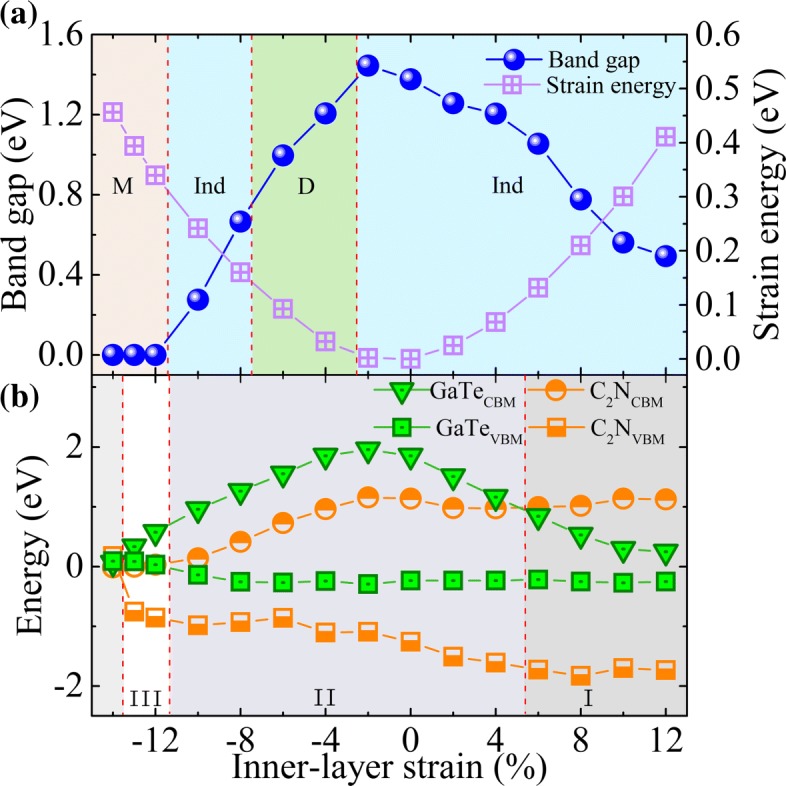
**a** In-plane biaxial strain effects on the bandgap and strain energy of the GaTe/C_2_N heterostructure.The mistyrose, blue, and green regions represent the metal (M), Ind and D bandgap ranges, respectively. **b** The evolutions of the band-edge positions of the sublayers in heterostructure as a function of the in-plane biaxial strain. The I, II, and III regions correspond to type-I, -II, and -III band alignments, respectively

Finally, we turn to exploring the effect of in-plane biaxial strains, which is simulated by changing the crystal lattice parameter and calculated by *ε*=(*a*−*a*_0_)/*a*_0_, where *a* and *a*_0_ are the lattice constants of the strained and pristine structures, respectively. To guarantee the in-layer biaxial strains considered are within the range of elastic response, we first examine the strain energy per atom, *E*_s_= (*E*_strained_−*E*_unstrained_)/*n*, with *n* being the number of atoms in the unit cell. The calculated strain-energy curve (see Fig. 6a (right *y*-axis)) shows a characteristic of the quadratic function, indicating that all the strains considered are within the elastic limit and, therefore, are fully reversible. The bandgap evolution under various biaxial strains is given in Fig. 6a. One can see that the bandgap reaches its maximum value (∼1.45 eV) under the strain of about − 2%. At *ε* = − 12% the system undergos a semiconductor to metal transition, implying tunable conductive and transport properties of this heterostructure. Meanwhile, an interesting indirect-direct-indirect (Ind-D-Ind) bandgap transitions are found at *ε*≃− 3% and − 8%, respectively. These transitions are derived from the strain-induced band-energy shifts at different k-points (see the Additional file 1 for detail: Figure S7). The Ind-D transition and the changes in electronic structure due to strain may enhance the optical absorption [47]. In Fig. 3, we compare the optical absorptions of the GaTe/C_2_N heterostructures under strains of ± 6%, where their bandgaps are nearly the same. The results show that biaxial strains red-shift the optical spectra in the range of visible light, being consistent with the decreased bandgap discussed above. Interestingly, a − 6% strain leads to a significantly enhanced optical absorption in the region of [1.60–2.65 eV]. Furthermore, it is also found that strain can change the band alignment. As shown in Fig. 6b and Additional file 1: Figure S7, for *ε*≥+ 6%, the CBM of the GaTe sublayer shifts downwards and becomes the CBM of the heterostructure. As a result, the energies of the CBM and VBM in the GaTe sublayer are straddled by those in the C_2_N sublayer, leading to a transition from the type II to the type I. Here, we note that the CBM and VBM of the GaTe sublayer approach each other under large tensile strains and form a very small bandgap while those of the C_2_N sublayer have only a minor change. This behavior can be understood by first considering the strain effects on the electronic structures of the two isolated monolayers. Previous calculations showed that the bandgap of GaTe monolayer is much more sensitive to large tensile strains than that of C_2_N monolayer: Under large tensile strains, the former will become very small while the latter remains [11, 16]. This may be due to the buckling structure of GaTe, which is affected more significantly by in-plane strains. Since the overall interlayer interactions in the heterostructure is weak, mainly the vdW and the electrostatic interactions which have only minor effects on the bandgap, the behaviors of the two monolayers under large tensile strains are preserved in the GaTe/C_2_N heterostructure. In addition, for *ε*≥−12%, both the CBM and VBM of the GaTe sublayer become higher than those of the C_2_N sublayer, and thus, the type III band alignment is formed. However, when the compressive strain is further increased to be larger than − 13%, this type III band alignment is broken, where the C_2_N sublayer will become metallic. In a word, the strain can engineer effectively the type and value of the bandgap and band alignment of the GaTe/C_2_N heterostructure. This will be useful to design multi-functional high-performance electronic and optoelectronic devices.

## Conclusions

In summary, by performing first-principles hybrid DFT calculations, we have investigated systematically the strain-dependent structural, electronic, and optical properties of the GaTe/C_2_N heterostructure. It is predicted to be an indirect-gap semiconductor showing improved optical absorptions in the visible-UV range compared to its components. The type II band alignment and intrinsic built-in electric field inhibit the energy-wasted recombination of the photogenerated carriers and thus enhance the performance of optoelectronic devices. In particular, large normal tensile strains can make the system suitable for water splitting at certain pH. By studying the absorption and decomposition behaviors of a water molecule on the C_2_N sublayer in the heterostructure, we find that the absorption of H_2_O and the formation of H_2_ on the C_2_N surface are all energetically favorable, which is beneficial for photocatalytically producing hydrogen gas. In-plane compressive strains will induce the Ind-D-Ind and semiconductor-metal transitions, whereas in-plane tensile strains will induce the type II to type I or type III transition. These results demonstrate that the GaTe/C_2_N heterostructure has great potential in applications of multi-functional optoelectronic devices.

## Additional file

Additional file 1 **Figure S1**. Band structures of the GaTe and C_2_N monolayers; **Figure S2**. Phonon spectrum and MD simulations of GaTe/C_2_N heterostructures; **Figure S3**. Band structures of the of GaTe/C_2_N heterostructures; **Figure S4**. The plane-averaged charge density difference of the heterostructure; **Figure S5**. The effect of normal strain on the interlayer interaction **Figure S6**. Projected band structures of the heterostructures under the various normal strains; **Figure S7**. Projected band structures of the heterostructures under the various in-plane strains. (PDF 3733 kb)

## Abbreviations

2D: Two-dimensional
CBM: Conduction band minimum
CBO: Conduction band offset
DFT: Density functional theory
HSE06: Hybrid Heyd-Scuseria-Ernzerhof
PBE: Perdew-Burke-Ernzerhofer
PDOS: Partial density of states
PTMCs: Post transition metal chalcogenides
VBM: Valence band maximum
VBO: Valence band offset
vdW: van der Waals
