# Supplementary material for: Strain-Tunable Electronic Properties and Band Alignments in GaTe/C2N Heterostructure: a First-Principles Calculation
Source: Nanoscale Res Lett. 2018 Sep 26;13:300. doi: 10.1186/s11671-018-2708-x (PMC6158146; doi:10.1186/s11671-018-2708-x)
Supplement: Supplementary file 1 — Figure S1. Band structures of the GaTe and C2N monolayers; Figure S2. Phonon spectrum and MD simulations of GaTe/C2N heterostructures; Figure S3. Band structures of the of GaTe/C2N heterostructures; Figure S4. The plane-averaged charge density difference of the heterostructure; Figure S5. The effect of normal strain on the interlayer interaction Figure S6. Projected band structures of the heterostructures under the various normal strains; Figure S7. Projected band structures of the heterostructures under the various in-plane strains. (PDF 3733 kb) [file 11671_2018_2708_MOESM1_ESM.pdf]

Supporting Information for

Strain tunable electronic properties and band alignments in GaTe/C_2N heterostructure: A first principles calculation

Xiao-Hua Li[1], Bao-Ji Wang[1]*, Xiao-Lin Cai[1], Wei-Yang Yu[1], Ying-Ying Zhu[1], Feng-Yun Li[1], Rui-Xia Fan[1], Yan-Song Zhang[1], and San-Huang Ke[2]*

[1]School of Physics and Electronic Information Engineering, Henan Polytechnic University, Jiaozuo, People's Republic of China

[2]MOE Key Laboratory of Microstructured Materials, School of Physics Science and Engineering, Tongji University, Shanghai, People's Republic of China

*Corresponding authors: wbj@hpu.edu.cn and shke@tongji.edu.cn

1. Band structures of the GaTe and C_2N monolayers.

2. Phonon spectrum and MD simulations of GaTe/C_2N heterostructures.

3. Band structures of the of GaTe/C_2N heterostructures.

4. The plane-averaged charge density difference of the heterostructure.

5. The effect of normal strain on the interlayer interaction.

6. Projected band structures of the heterostructures under the various normal strains.

7. Projected band structures of the heterostructures under the various in-plane strains.

1. Band structures of the GaTe and C_2N monolayers.

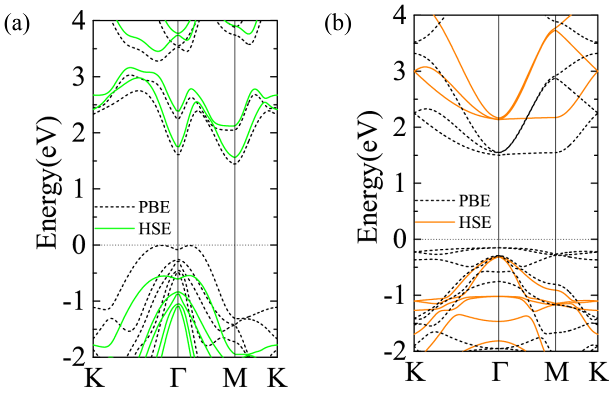

Fig. S1. Band structures of the GaTe (a) and C_2N (b) monolayers. All results are calculated with PBE and HSE06 functionals.

2. Phonon spectrum and MD simulations of GaTe/C_2N heterostructures.

A large (2 × 2 × 1) supercell is built to calculate the force constant and then to obtain their phonon spectra. The same supercell is also used for the molecular dynamics (MD) simulation to check their thermal stability, where the constant temperature (NVT) MD simulation is performed at 300 K for 6 ps using a time step of 2 fs.

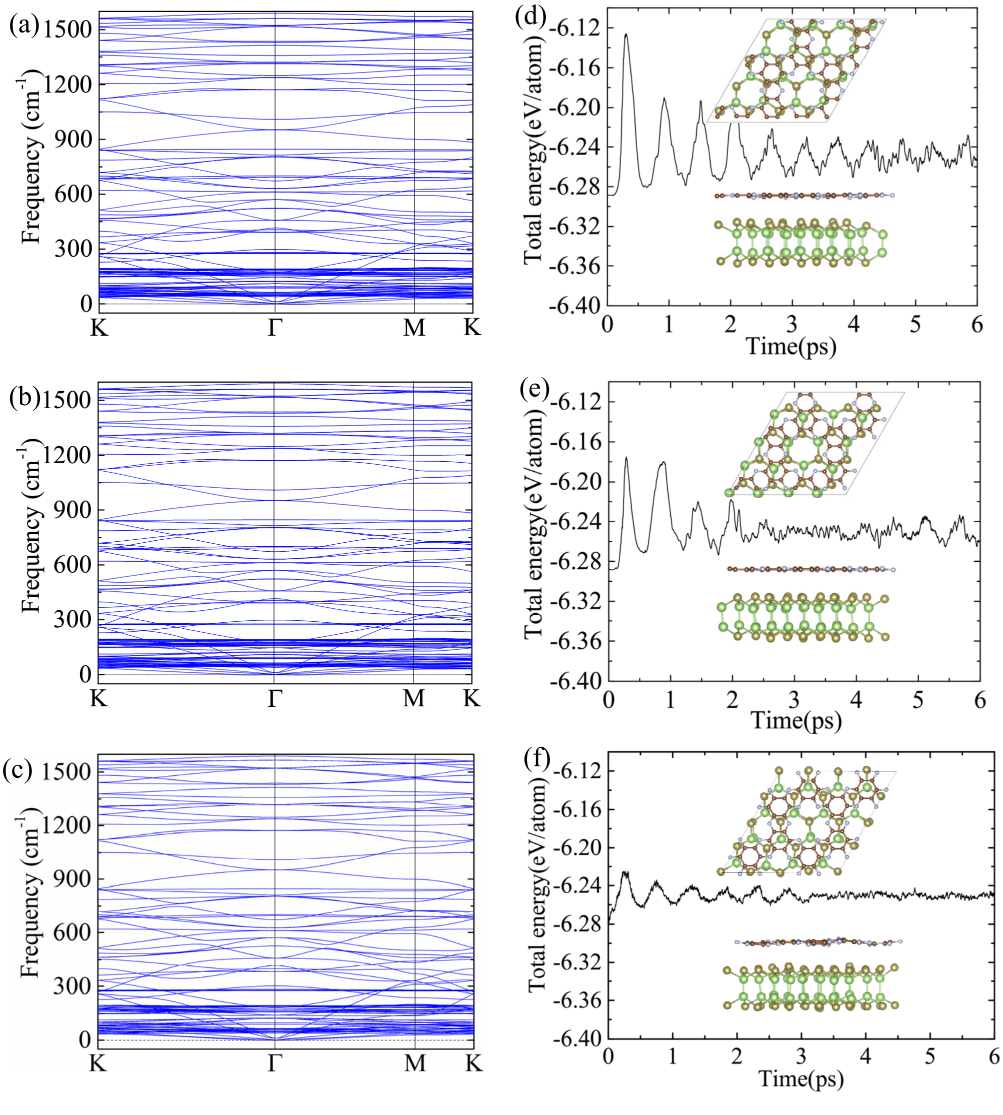

Fig. S2. Phonon dispersion curves of GaTe/C_2N heterostructures with (a) α-, (b) β-, and (c) γ-stacking configuration, respectively and (d, e, f) corresponding variation of the total energy in the molecules dynamic simulation. The ultima structures are also displayed (see inserts).

3. Band structures of the of GaTe/C2N heterostructures.

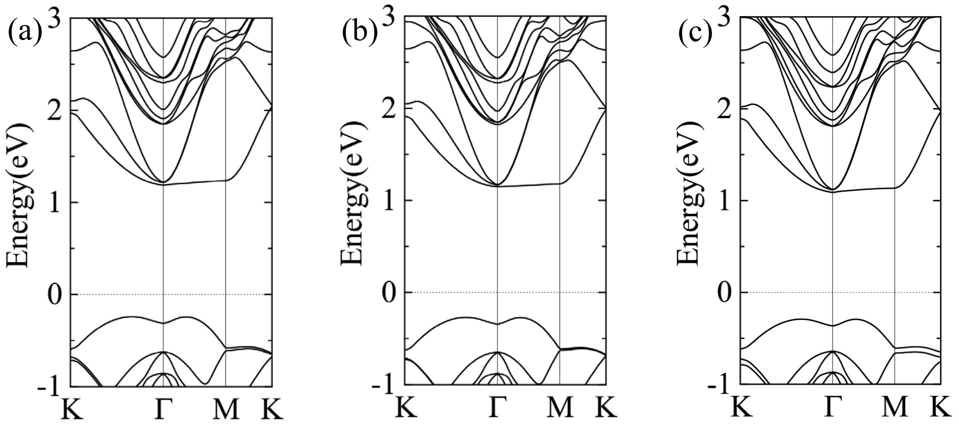

Fig. S3. Band structures of GaTe/C2N heterostructures with (a) α-, (b) β-, and (c) γ-stacking configuration, respectively.

4. The plane-averaged charge density difference of the heterostructure.

The plane-averaged charge density difference along z-direction normal to the heterostructure is calculated as $\Delta\rho(z) = \rho_H(z) - \rho_{GaTe}(z) - \rho_{C_2N}(z)$, where $\rho_H(z)$, $\rho_{GaTe}(z)$ and $\rho_{C_2N}(z)$ are the plane-averaged charge densities of the heterostructures, the pristine monolayer GaTe and C_2N, respectively. The amount of transferred electrons up to z is given by $\Delta Q(z) = \int_{-\infty}^{z} \Delta\rho(z')\, dz'$. Consequently, the total number of electrons transferred between GaTe and C_2N layers is determined by the value of $\Delta Q(z)$ at the GaTe/ C_2N interface.[1]

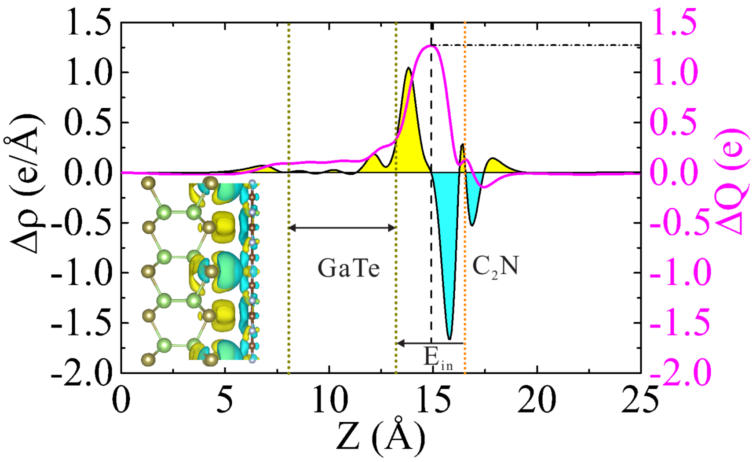

Fig. S4. The plane-averaged charge density difference ρ(z) (black) and the amounts of transferred charge Q(z) (pink) along the normal direction of the heterostructure. The inset is the 3D isosurface of the charge density difference. The yellow and cyan areas represent electrons accumulation and depletion, respectively. The vertical black dashed line denotes the GaTe/C_2N interface. E_{in} is the built-in electric field.

5. The effect of normal strain on the interlayer interaction

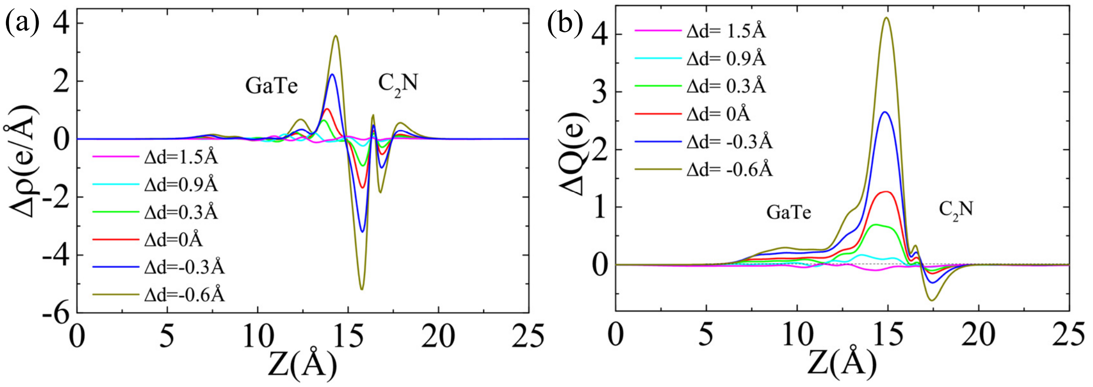

FIG. S5. (a) Plane-averaged charge density differences of the GaTe/C_2N vdW heterostructures with different normal strains and (b) the corresponding amount of transferred charge ΔQ along the normal direction of the surface. Here, the amount of transferred electrons from the C_2N to GaTe layer increases with the decrease in interlayer spacing, which suggests an enhanced interlayer interaction.

6. Projected band structures of the heterostructures under the various normal strains.

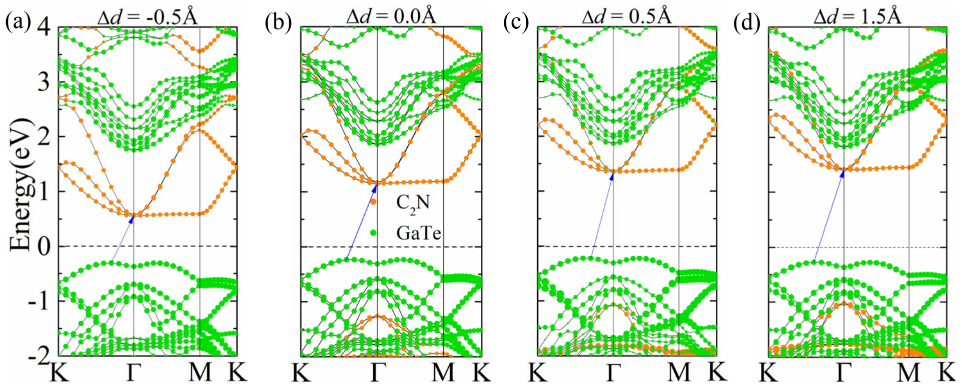

Fig. S6. Projected band structures of the heterostructures with normal strains of (a) -0.5, (b) 0.0, (c) 0.5, and (d) 1.5 Å. All results are calculated with HSE06 functional.

7. Projected band structures of the heterostructures under the various inner-layer strains.

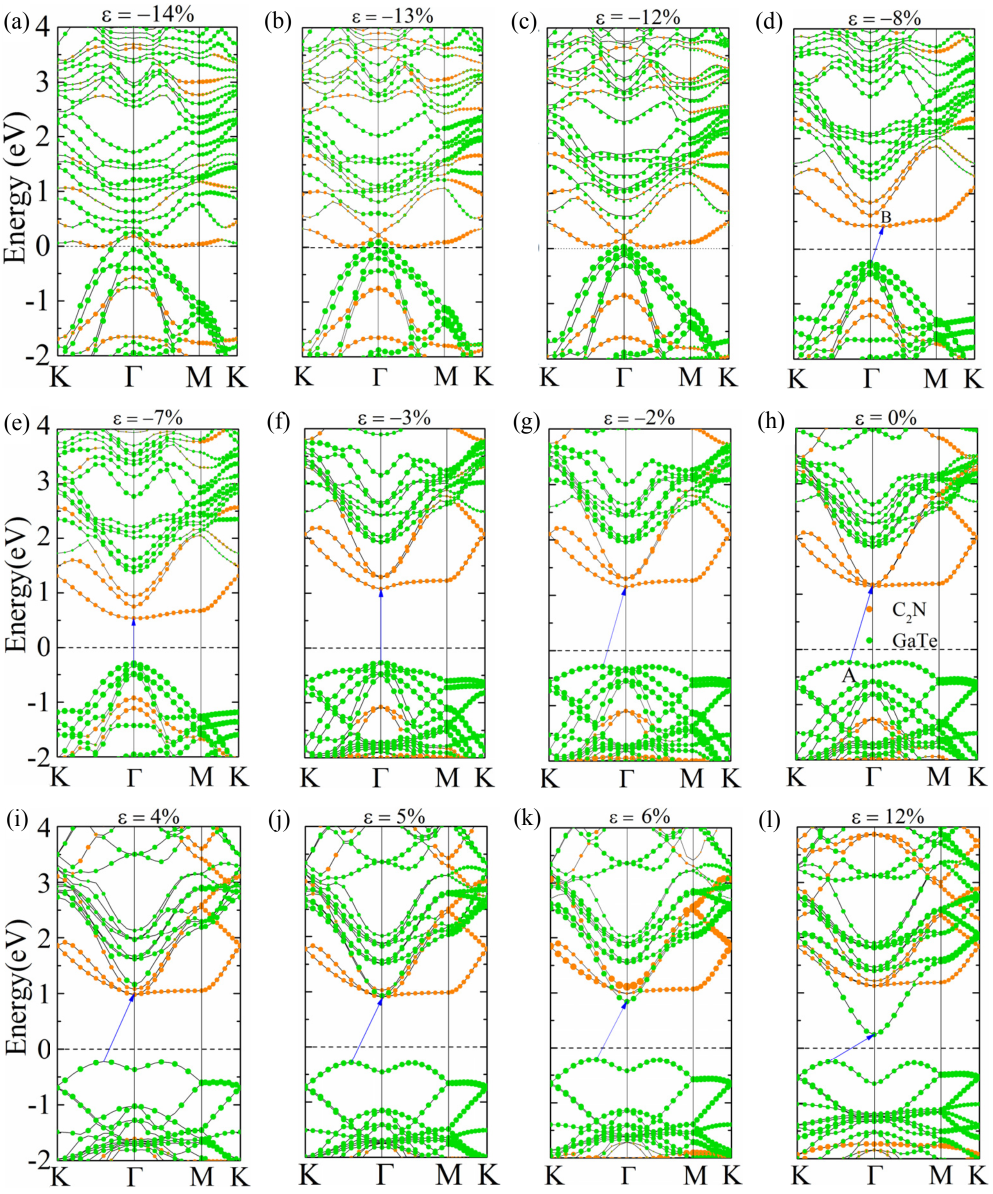

Fig. S7. Projected band structures of the heterostructures with in-plane strains of (a) -14%, (b) -13%, (c) -12%, (d) -8%, (e) -7%, (f) -3%, (g) -2%, (h) 0%, (i) 4%, (j) 5%, (k) 6%, (l) 12%. The results are calculated by HSE06 functional.

References

[1] B. J. Wang, X. H. Li, X. L. Cai, et al., *J Phys. Chem. C*, 2018, **122**, 7075.
